# Indirect traumatic optic neuropathy

**DOI:** 10.1186/s40779-016-0069-2

**Published:** 2016-01-11

**Authors:** Eric L. Singman, Nitin Daphalapurkar, Helen White, Thao D. Nguyen, Lijo Panghat, Jessica Chang, Timothy McCulley

**Affiliations:** Wilmer Eye Institute at Johns Hopkins Hospital, Baltimore, Maryland USA; Whiting School of Engineering, Johns Hopkins University, Baltimore, MD USA; Director of Informatics and Information Management, Vision Center of Excellence [VCE], Office of the Assistant Secretary of Defense for Health Affairs [ASD-HA], United States Department of Defense [DOD], Walter Reed National Military Medical Center, Bethesda, MD USA

**Keywords:** Indirect trauma, Optic nerve, Blunt, Blast, Neuropathy, Biomechanics, Virtual model

## Abstract

Indirect traumatic optic neuropathy (ITON) refers to optic nerve injury resulting from impact remote to the optic nerve. The mechanism of injury is not understood, and there are no confirmed protocols for prevention, mitigation or treatment. Most data concerning this condition comes from case series of civilian patients suffering blunt injury, such as from sports- or motor vehicle-related concussion, rather than military-related ballistic or blast damage. Research in this field will likely require the development of robust databases to identify patients with ITON and follow related outcomes, in addition to both in-vivo animal and virtual human models to study the mechanisms of damage and potential therapies.

## Background

Indirect traumatic optic neuropathy (ITON) is a condition in which a patient suffers head trauma and is found to have reduced vision and an afferent pupillary defect despite a normal acute slit lamp examination and normal acute magnetic resonance imaging (MRI) and computer tomography (CT) of the optic nerve and canal. ITON differs substantially from direct traumatic optic neuropathy (DTON); in DTON, the optic nerve axons or vascular supply are directly damaged due to strain (as may be observed in an avulsion of the optic nerve), compression (as may be observed from edema or retro-orbital hemorrhage) or transection (as may be observed from a fracture in the optic canal). Patients suffering either ITON or DTON can experience profound vision loss after trauma. Prognosis is guarded in either situation; however, some patients with ITON may experience full recovery with no intervention. Few randomized controlled studies have explored therapies for ITON, and as discussed below, therapies employed to treat DTON appear to be ineffective for ITON. A computerized search of PubMed (performed October 28, 2015, search terms: indirect + trauma + optic + nerve) revealed 195 publications on this topic from 1905-present; notably, a similar search without the word “indirect” produced 4,495 entries. Most publications concerning ITON can be divided into studies of diagnosis, therapeutic approaches and pathogenesis. Additionally, the vast majority of publications report on civilian blunt injuries rather than blast or ballistic injuries that may be experienced more commonly by military personnel.

## Epidemiology

The incidence of ITON has not been directly reported in any large population. However, very good surveillance studies of traumatic optic neuropathy of any type have been reported for pediatric [1] and adult [2] populations in England. For both adults and children, the overall incidence of traumatic optic neuropathy is approximately 1/million. Notably, approximately 80 % of the patients were male, and the majority of cases suffered relatively minor head injuries with neither orbital nor skull fracture, suggesting that ITON may be more common than DTON. Visual outcomes ranged from no light perception (NLP) to normal. One study from India reported that 35 (or 27 %) of 129 consecutive cases collected between 1994–2006 suffered ITON following cranio-orbital injury in two-wheeler riders involved in road accidents [3]. Notably, these patients reportedly did not wear protective helmets, suggesting that helmets may offer protection. Case reports suggest that the location of the impact, specifically the face, is also a risk factor for ITON [4–7]. A retrospective study of 379 consecutive patients who underwent repair for facial fractures in the US reported that blindness developed in at least one eye in 21 patients (6 %), 5 of whom had ITON (1.3 %) [8]. There has been a call to create a registry specifically for ITON [9]; however, no such registry has been created. The United States Department of Defense (DoD) Vision Center of Excellence (VCE), in collaboration with Veteran Health Affairs, developed a robust registry: the Defense and Veterans Eye Injury and Vision Registry (DVEIVR). The vision registry is composed of active duty service members and veterans with eye trauma and vision dysfunction/damage to the visual system. This initiative of the VCE consists of clinical and historical data starting on 9/11/2001 and provides a longitudinal analysis of patients with trauma and other ocular and visual pathway injuries [10]; the authors are currently exploring this registry to determine if it may yield a cohort of patients with traumatic optic neuropathy from any cause in general and ITON in particular.

## Diagnosis

By definition, ITON is a condition in which injury to the optic nerve can be clinically confirmed, i.e., patients demonstrate reduced visual acuity, color vision, and/or visual field, as well as a relatively afferent pupillary defect. Notably, this latter finding may not be present in bilateral cases. Direct ophthalmoscopy of the nerve is also expected to appear normal, though optic atrophy or pallor are expected to develop. Automated visual field testing should be offered; however, the vision of subjects may be too poor to glean useful results. In most cases, testing with visual evoked potentials (VEP) is not needed to establish the diagnosis. However, in questionable cases, VEP may provide confirmatory data. VEP may also have predictive value; patients with better responses on VEP may be more likely to regain some or all of their vision [11, 12]. VEP has also been employed in the emergency setting to guide decisions to offer rescue treatment [13]. One case reported a patient with ITON who was evaluated with scanning laser polarimetry, which indicated that the retinal nerve fiber layer acutely thickened and then progressively thinned for 3 months [14]. This trend was supported by results from a Chinese study employing ocular coherence tomographic (OCT) evaluation in 54 patients with ITON. Retinal nerve fiber layer thickness ultimately decreases in these patients; however, in those eyes with the worst vision (no light perception [NLP]), there was a slight increase in thickness in the acute (first 2 weeks) post-injury period [15]. Additionally, this study explored hemodynamic parameters and demonstrated that the peak systolic velocity of blood flow within the central retinal artery was significantly increased in ITON patients regardless of whether their visual acuity was NLP or better than NLP.

CT and plain radiographic findings in ITON would, by definition, exclude fractures in the optic canal because they would be suggestive findings of DTON. However, patients with ITON may suffer other skull fractures. A study of 39 patients with both ITON and peri-orbital fractures indicated that posterior orbital fracture is associated with poorer visual outcomes than those with anterior fractures, suggesting that CT may help in guiding decisions to offer therapy [16]. MRI, particularly the diffusion tensor imaging (DTI) technique, has been applied to the study of ITON. In a study of 28 patients, no differences were noted between the injured eye and the normal contralateral eye in the first week after injury. Reduced fractional anisotropy appeared in the injured eye by the second week, a trend that continued after one month [17]. These results indicate that MRI may be useful to follow the patient but may not be as helpful in the initial diagnosis.

## Treatment

No therapy has been confirmed to effectively treat ITON, but observation is considered an acceptable option.

Observational studies of ITON have demonstrated wide variability in visual function, the rate of recovery and long-term outcomes. Most patients in the pediatric TON surveillance study improved or at least did not worsen from baseline visual acuity [1]. Recovery usually occurs within the first month after injury [18] but can occur later at 8 or 12 weeks [12].

Two pilot studies exploring the efficacy of intravenous (IV) erythropoietin have been published [19, 20]. In both cases, the drug was administered within 2–3 weeks of onset, and in both cases, the treated cohort demonstrated improved best corrected visual acuity. Notably, the rationale for this treatment was that erythropoietin may provide neuro-protection and support axonal growth [21–24].

Corticosteroids have also been offered to patients for ITON. A Cochrane review from 2013 found one double-masked, placebo-controlled and randomized study in which high-dose IV corticosteroids were offered within 1 week of the injury causing ITON; there was no significant benefit over observation [25]. This finding was supported by an even more recent review of multiple electronic databases [26]. Importantly, a large multicenter study (MRC CRASH) indicated that high-dose corticosteroids should not be routinely offered to patients suffering a head injury due to an elevated risk of death [27].

A 2010 study investigated the potential additive effect of providing levodopa-carbidopa to improve the visual outcomes of patients with indirect traumatic optic neuropathy (ITON) [28]. The rationale behind this study was that steroids should be offered because they were considered a mainstay of treatment and that the neuro-protective effect of levodopa may enhance outcomes [29]. This randomized, double-blind, placebo-controlled study was completed on 32 patients with ITON within 6 days after trauma. All patients received high-dose intravenous methylprednisolone, and levodopa was also administered to 16 patients. Visual acuity significantly improved in the levodopa group but not the placebo group. Nine patients in the levodopa group and 1 in the placebo group experienced improvement in visual acuity; no patients worsened over time. Recognizing that the evidence supporting the efficacy of corticosteroids is insubstantial, one must wonder whether the levodopa-carbidopa itself may provide a benefit. The neuro-protective effects of levodopa have also been suggested in more recent studies [30].

Optic canal decompression represents the primary surgical therapy attempted in treating ITON. A number of retrospective studies provided anecdotal data suggesting that this surgery can be effective even if offered after a few weeks of observation or medical therapy [18, 31–37]. However, a Cochrane review in 2005 found no strong evidence of the efficacy of canal decompression but did find risks of vision loss, cerebrospinal fluid (CSF) leak and meningitis [38]. Prior to that, the International Optic Nerve Trauma Study similarly concluded that neither steroid therapy nor decompression showed clear benefits [39].

Transcorneal electrical stimulation (TES) has been reported to maintain more normal morphology and increase the survival of rat retinal ganglion cells after nerve crush [40], to promote axon survival and regeneration [41], and to support improved function of the optic nerve [42]. The rationale for offering TES is that earlier studies suggested that it may have stimulated the production of neuro-protective substances. TES has been offered to 5 humans suffering from traumatic optic neuropathy, 4 of whom reportedly experienced visual improvement [43]. Notably, this publication specified neither the mechanism of injury causing the traumatic optic neuropathy nor how that diagnosis was made. A later study reported that TES increases choroidal blood flow [44]; this was postulated to be neuro-protective. TES may warrant further exploration due to its safety and the data supporting its positive effect on photoreceptors and ganglion cells in animal experiments [45].

Notably, one reported case series found that acupuncture, a traditional Chinese medicine, was successfully applied to treat ITON [46]. Acupuncture has also been reported to help patients with ischemic optic neuropathy [47], though it has not been shown to be successful in treating glaucoma [48]. Until other therapies are proven beneficial, it appears reasonable to consider further studying the potential of acupuncture given its very low risk.

Research on novel neuro-protective medical therapies for optic nerve injury, particularly against glaucoma [49–54], optic neuritis [55, 56] and direct nerve trauma [57–62], is flourishing. Additionally, efforts are aimed not only at sparing retinal ganglion cells but also at regenerating their axons [63–66]. These therapies are in varying stages of development but are not currently readily available to practitioners.

## Etiology

Studies suggest that the biomechanical response of the cranial contents to traumatic loads is an important aspect in understanding the etiology of ITON. A seminal and frequently referenced study using holographic interferometry on human skulls reported that frontal loading results in the deformation of the ipsilateral orbital roof near the optic foramen [67]. One may suggest that this deformation could damage not only the supporting vasculature of the optic nerve but could also cause shear stresses to the nerve, particularly where the nerve enters the canal.

A more recent anatomic study of cadaveric orbits and optic nerves with the intent of identifying possible etiologies for ITON was reported in 2002 [68]. Forty-one specimens were analyzed via light, polarization, immune-histochemical and scanning electron microscopy. Delicate anastomoses were reported to run between the dura and pia in the optic canal, and potential mechanisms of optic nerve injury include the disruption of the blood supply, pressure from microhematomas and edema, and direct shearing injury to axons.

Diffuse axonal damage should also be considered as a mechanism underlying ITON. Diffuse axonal damage due to injurious inertial force to the head has been associated with poor neurological outcomes. Rapid deformations of axons in the white matter tracts of the brain can lead to damage to the axonal cytoskeleton and the impairment of axoplasmic transport. Subsequent swelling and calcium entry into damaged axons can lead to further dysfunction and physical breakage and additional neuropathological changes in the brain tissue [69]. There is a need to improve the current understanding on the connection between the biomechanics and pathophysiology associated with axonal trauma. In vivo experiments that stretched an optic nerve in guinea pigs have provided tissue-based mechanical criteria for axonal injury using a measure of strain, which was defined as the ratio of the amount of extension over the unstretched length [70]. Integrating these experimentally-derived criteria with high-fidelity head anatomy from MRI and DTI data within a physics-based framework have enhanced computational models of traumatic brain injury (TBI) [71]. For example, Cirovic et al. [72] performed a finite element analysis of the human globe and orbit to study the passive mechanisms of eye restraint during head impact trauma; this study explored ocular changes that may be observed in shaken baby syndrome. Modeling has been applied to understand the biomechanics of glaucomatous injury to the optic nerve [73–78], compressive injury to the chiasm [79], blunt injury to the optic nerve [80] and blast injury to the globe and orbit [81–83]. Data from trauma modeling in a virtual head and orbit suggest that frontally applied forces, even of low impact, propagate toward the optic foramen, supporting the notion that ITON is more likely to result from facial injuries than from injuries to other locations on the skull. Despite these advances, the etiology of ITON remains unclear. A holistic model of the orbit, including both bone and soft tissue elements, may bring greater understanding to this blinding illness and permit strategies for prevention and mitigation. Additionally, a better model would enable studies not only of blunt injury commonly experienced by civilians but also ballistic and blast injury in warfare.

## Conclusions

Little is known with certainty about the etiology of indirect traumatic optic neuropathy. This sight-threatening condition may be a relatively common comorbidity of blunt craniofacial head trauma. Unfortunately, there are neither proven strategies for prevention, mitigation or cure, nor are there satisfactory laboratory models to explore this condition. The authors strongly support the development and analysis of clinical databases to better understand the natural history of this condition. Further, the authors support efforts to create models, both virtual and real, to clarify the biomechanics of ITON.

## Abbreviations

CSF: cerebrospinal fluid
CT: computed tomography
DOD: Department of Defense (United States)
DTI: diffusion tensor imaging
DTON: direct traumatic optic neuropathy
DVEIVR: Defense and Veterans Eye Injury and Vision Registry
ITON: indirect traumatic optic neuropathy
IV: intravenous
MRI: magnetic resonance imaging
NLP: no light perception
OCT: ocular coherence tomography
TBI: traumatic brain injury
TES: transcorneal electrical stimulation
VCE: Vision Center of Excellence (of the US Dept. of Defense)
VEP: vision evoked potential
